# Patient-Derived Xenograft Models to Improve Targeted Therapy in Epithelial Ovarian Cancer Treatment

**DOI:** 10.3389/fonc.2013.00295

**Published:** 2013-12-04

**Authors:** Clare L. Scott, Marc A. Becker, Paul Haluska, Goli Samimi

**Affiliations:** ^1^Walter and Eliza Hall Institute of Medical Research, Royal Melbourne Hospital and The Royal Women’s Hospital, Melbourne, VIC, Australia; ^2^Division of Medical Oncology, Mayo Clinic, Rochester, MN, USA; ^3^The Kinghorn Cancer Centre, St Vincent’s Clinical School, Garvan Institute of Medical Research, Sydney, NSW, Australia

**Keywords:** ovarian cancer, patient-derived xenografts, pre-clinical models, targeted therapy, clinical trials

## Abstract

Despite increasing evidence that precision therapy targeted to the molecular drivers of a cancer has the potential to improve clinical outcomes, high-grade epithelial ovarian cancer (OC) patients are currently treated without consideration of molecular phenotype, and predictive biomarkers that could better inform treatment remain unknown. Delivery of precision therapy requires improved integration of laboratory-based models and cutting-edge clinical research, with pre-clinical models predicting patient subsets that will benefit from a particular targeted therapeutic. Patient-derived xenografts (PDXs) are renewable tumor models engrafted in mice, generated from fresh human tumors without prior *in vitro* exposure. PDX models allow an invaluable assessment of tumor evolution and adaptive response to therapy. PDX models have been applied to pre-clinical drug testing and biomarker identification in a number of cancers including ovarian, pancreatic, breast, and prostate cancers. These models have been shown to be biologically stable and accurately reflect the patient tumor with regards to histopathology, gene expression, genetic mutations, and therapeutic response. However, pre-clinical analyses of molecularly annotated PDX models derived from high-grade serous ovarian cancer (HG-SOC) remain limited. *In vivo* response to conventional and/or targeted therapeutics has only been described for very small numbers of individual HG-SOC PDX in conjunction with sparse molecular annotation and patient outcome data. Recently, two consecutive panels of epithelial OC PDX correlate *in vivo* platinum response with molecular aberrations and source patient clinical outcomes. These studies underpin the value of PDX models to better direct chemotherapy and predict response to targeted therapy. Tumor heterogeneity, before and following treatment, as well as the importance of multiple molecular aberrations per individual tumor underscore some of the important issues addressed in PDX models.

## Introduction

Cell lines and archival tumor tissue have provided a platform for discovery and validation of novel therapeutic targets in epithelial ovarian cancer (OC). However, despite increasing evidence that precision therapy targeted to the molecular driver(s) of a tumor has the potential to impact overall survival (1), patients with high-grade epithelial OC are currently treated with a “one-size fits all” approach, without consideration of molecular phenotype or biomarkers of response that could better inform treatment. Pre-clinical models to predict those patients who will benefit from targeted therapy are imperative to implement effective precision therapy strategies. Classic cell line-derived xenograft models have provided invaluable mechanistic insight toward the key signaling pathways and oncogenic drivers of OC tumorigenesis, malignant progression, and chemotherapeutic resistance. The translational potential of models, generated from either human OC, many years ago, with scant histo-pathologic data about the source OC tumor from which they were derived or from human ovarian surface epithelial (OSE) cell lines, remains questionable (2). In both cases, the cell lines used to generate xenografts have been expanded *in vitro*, and as such, have likely acquired significant alterations in morphology, motility, and proliferation that do not necessarily reflect the physiologic state of the tumor (3). More importantly, recent evidence suggests that the ovarian surface may not be the origin of “ovarian cancer” (4, 5).

The majority of epithelial OC are serous in sub-type (50% of OC) and display Fallopian tube-like or “endosalpingeal” characteristics. Endometrioid OC (20% of OC) and mucinous OC (10% of OC) represent additional epithelial sub-types displaying features of epithelia from other Mullerian tract (developmental female genital tract) organs, resembling endometrial and endocervical epithelia, respectively (6). Two main phenotypic groupings of human EOC have been described. Type I EOC includes low-grade, mucinous, and clear cell cancers, with progression identifiable from adenoma-borderline-cancer. Type II EOC comprises lethal, high-grade serous (HG-SOC), endometrioid, and undifferentiated EOC. Although previously thought not to have recognizable precursor lesions (7), the current consensus indicates an association with early lesions being found in the distal Fallopian tube in carriers of *BRCA1/2* mutations (5, 8). The Fallopian tubes are derived from the Mullerian ducts (also of mesodermal origin) and consist of muscular, ciliated, and secretory epithelia (9). Fallopian tube cancer and primary Peritoneal cancer (the latter is derived from the coelomic epithelium, as is the OSE), behave in a clinically similar fashion to serous EOC and while often studied together, distinct molecular differences are evident (10, 11).

The OSE has long been postulated to be the source of putative cancer initiating cells for epithelial “ovarian” cancer (12), with an alternative origin postulated to derive from the distal ends of the Fallopian tube and malignant or pre-malignant cells migrating to and settling on contiguous OSE (5, 8). A proportion of malignant lesions originate in the Fallopian tube and may potentially metastasize to the OSE (13). The OSE persists as a single layer of squamous to cuboidal epithelium that covers the ovary (14), is derived from coelomic epithelium (of mesenchymal origin), has a basement membrane and, unusual for a surface epithelium, can undergo epithelial-mesenchymal transition (EMT) (15). The OSE has been described as a facultative stem cell niche, with cells retained within this niche maintaining pluripotency and expressing markers typical of a stem cell-like quiescent state (12). However, it is possible that the OSE provides a suitable “niche” for the development of “ovarian” cancer and that the majority of HG-SOC in fact derive from secretory cells from the fallopian tube (16). Many reports of OC xenografts and patient-derived xenografts (PDXs) have mixed together OC sub-types, which may have very different implications for cell of origin or treatment approaches. By not taking into account sub-type, the likelihood of deriving useful information is greatly diminished.

Furthermore, traditional *in vivo* pre-clinical models do not accurately recapitulate the complexity and heterogeneity of patient tumors (17). As each tumor’s molecular phenotype impacts prognosis and response to treatment, detailed genomic annotation of each xenograft is necessary for comprehensive evaluation of targeted therapies. Xenografts derived from a cell line originating from OC have to undergo extensive selection. More often these lines reflect the *in vitro* culture system and are devoid of the complex pathology and molecular attributes of the original patient tumor. A compelling example of this discordant phenomenon was reported in a study involving 41 cell lines, each of which rarely contained *BRCA1* and/or *BRCA2* mutations (18). The authors concluded that the use of these cell lines for xenograft studies would not accurately reflect the patient population. Moreover, xenografts derived from cell lines cultured from potentially irrelevant tissues (e.g., non-surface epithelium), may be even more flawed as models of human HG-SOC.

Xenografts may be derived directly from patient tissue without prior *in vitro* culture (PDXs), in which tumor tissue excised at the time of surgery is immediately transplanted into immune-deficient mice. Importantly, digestion of tumor material using protocols known to involve harsh cell dissociation buffers may inadvertently strip the cell surface of molecules integral toward *in vivo* cell–cell interactions. Alternatively, methods such as mincing of tumor fragments or the use of whole fragments may more closely model the heterogeneity of clinical disease. PDXs derived from non-digested OC can provide extremely flexible models for pre-clinical analysis of novel therapeutics (19). Primary OC and resultant serial PDX can be histologically assessed for known diagnostic and prognostic markers and characterized by molecular techniques including genome sequencing. These PDX models can therefore be extensively annotated and serve as powerful models for pre-clinical studies of targeted therapeutic strategies, thus bridging the gap between lab bench discoveries and clinical translation. As such, there has been an increase in characterization and application of PDX models for drug screening across a range of cancers [reviewed in Ref. (17)].

Thus, major concerns regarding OC PDX literature to date are as follows: numerous papers lack detail regarding histologic sub-type, molecular phenotype, a detailed description of the methods used to generate and maintain the PDX, limited genomic characterization has been performed (e.g., CGH or CNV analysis), and the stability of various phenotypes over successive generations is noteworthy. As a result, a substantial barrier to the study of OC is the paucity of translationally (e.g., transient *in vitro* primary cell lines) and clinically (e.g., archived tissues from retrospective analyses) relevant models, thereby highlighting the salient need for an alternative, clinically relevant means to rapidly translate results from bench-to-bedside. The development of personalized PDX models, with each patient having a PDX generated across her disease progression (primary tumor, metastasis, recurrence) and stage of treatment (prior to treatment, at relapse), with availability of source biospecimens (germline DNA, serum, frozen, and FFPE tissue, etc.) and prospective clinical annotations could overcome many of the current hurdles (e.g., the dependence on isolation/digestion and subsequent amplification *in vitro* prior to establishment and testing in animals). These PDX models recapitulate primary patient tumors (e.g., formation of bowel metastases, obstruction, ascites, etc.), reproducibly engraft, retain the molecular and gross phenotypic characteristics of the donor OC patient, can be accurately monitored for tumor detection and progression (e.g., gross tumor palpation, calipers, ultrasound-guided imaging, etc.) and represent a practical and highly translatable medium to study the effects of both standard chemotherapy and precision targeted therapeutics.

### Methodology

As previously noted, standard OC xenografts are derived from established, highly annotated, and widely available cell lines. While OSE models are commonly utilized and have become a mainstay workhorse to investigators, their uncommon origin (e.g., murine-derived) brings into question functional significance. For example, the ID8 cell line was originally developed as a syngeneic mouse model to study the early molecular and immune events related to ovarian carcinogenesis (20). As a result, greater attention toward patient-derived OC models has been expended.

Patient-derived xenograft (digested) have played a key role toward the study of the cancer stem cell (CSC) niche and identified tumor-initiating cells (TICs) as key players in primary patient ovarian xenografts (21). The frequency of TICs represents an intrinsic property of the primary patient tumor. However, the integrity of the TIC landscape (e.g., proportion of CD133 positive versus negative cells) is altered in PDX models. It is plausible that the extensive *ex vivo* digestion prior to PDX engraftment is the confounding source of TIC PDX discrepancies.

Patient-derived xenograft (fragments) are generated by sectioning of fresh tumor tissue and engrafting (1–2 × 1–3 mm^3^) pieces either subcutaneously or orthotopically into immuno-deficient mice (e.g., NOD-SCID IL2Rγ^−/−^). Engraftment rates for this generation (T1) range from 25 to 80% depending on tumor type (22), and growth usually takes 2–6 months. Once the T1 PDX tumor has reached ∼700–1500 mm^3^ (23), it is harvested and directly re-transplanted for expansion in later serial generations (T2, T3) which are used for *in vivo* drug response, biomarker studies and generating cell lines for additional drug response and molecular studies. Alternatively, the fresh patient tumor can be minced and cryo-preserved in DMSO for later thawing and transplantation, thus ensuring the renewability of the resource. For molecular comparisons, the original patient tumor and the PDX models can undergo extensive histo-pathological and genomic analysis. In addition, for OC PDX models, the T1 PDX can be analyzed for platinum response and homologous recombination (HR) activity, which are key clinical indicators of drug response.

### PDX models in ovarian cancer

Patient-derived xenograft models have been applied to pre-clinical drug testing and biomarker identification in a number of cancers including pancreatic cancer (24), NSCLC (25, 26), melanoma (27), breast cancer (28, 29), and prostate cancer (30). As reviewed by Tentler et al. comprehensive genomic analysis including sequencing, expression, and copy number, have demonstrated that PDX models maintain overall global gene expression and activity as the source tumor (17). In OC, PDX models have been developed that accurately reflect the patient tumor and have successfully been used to examine drug response and effects of targeted treatment (22).

Some of the earliest applications of OC PDXs in studies of drug response were reported by the Repasky group. They developed 20 different PDX models in severe combined immuno-deficient (SCID) mice. Histo-pathologic and *in situ* hybridization analyses were carried out to confirm similarity to the source tumor. While all implanted PDXs eventually formed tumors, 65% (13/20) of them reached 1–2 cm within 2–6 months and were further expanded. Three of the later generation PDX models developed metastases and two developed ascites, representing clinical progression of the disease (31). The group then applied their subcutaneous PDX models in two separate studies to examine the effects of IL-12 and Flt-3 ligand on ovarian tumor growth. Following engraftment, PDX mice were treated with either placebo or IL-12 (32), or placebo or Flt-3 ligand (33), and tumor volume was measured over time. Treatment with IL-12 or Flt-3 ligand resulted in decreased tumor growth compared to control-treated mice, with increased NK cells and necrosis in the tumors of IL-12 or Flt-3 ligand treated mice. These findings suggest an immunologic reaction in response to IL-12 and Flt-3 ligand, supporting their potential therapeutic roles in the treatment of OC (32, 33).

Ghamande et al. followed up these studies by examining the effect of CD40 ligand therapy, previously shown to decrease growth in OC cells, on CD40 receptor-positive PDX serous OC models (34). PDX mice with subcutaneous or intra-abdominal tumors were treated with vehicle or increasing concentrations of recombinant CD40 ligand and tumor growth was assessed over time. Tumor growth in both locations was decreased following as little as one cycle of treatment, regardless of concentration. In addition, once tumors were excised following treatment, histological analysis revealed disruption of tissue architecture and increased fibrosis and apoptosis, providing further insight into the mechanism of therapy. Furthermore, the authors utilized these PDX models to examine the effect of combination therapy using standard chemotherapeutic agents and CD40 ligand therapy, further demonstrating an augmented effect when both drugs were used in treatment of CD40-positive tumors (34). These studies highlight the utility of PDX models in evaluating drug efficacy and mechanism of action.

While a majority of HG-SOC patients initially respond to first-line treatment (generally, a platinum drug in combination with a taxane), a large proportion eventually relapse and develop platinum-resistant disease. OC PDX models can be useful for screening drug sensitivity, which in turn provides guidance for clinical management of the patient who presents with recurrent disease. Kolfschoten et al. established 15 subcutaneous OC PDX models and examined sensitivity to standard chemotherapy (35). They reported that response rates in the PDX models correlated with those in OC patients (e.g., 40% of PDXs responded to cisplatin while 48% of patients respond). As detoxification by glutathiones has been demonstrated to render cells resistant to platinum treatment, the authors also investigated the glutathione-based mechanisms involved in the development of resistance. They measured levels of glutathione and glutathione-related enzymes in the PDX models and related them to drug response. They identified a correlation between glutathione reductase activity and efficacy of chemotherapeutic agents cisplatin and cyclophosphamide, suggesting that glutathione-related enzymes may be useful as predictors of drug sensitivity (35). These findings speak to the value of PDX models for expanding *in vitro* findings of drug response and relating them to patient tumors.

Because HG-SOC patients frequently develop resistance to platinum-based chemotherapy, it is imperative to identify novel therapies with efficacy toward tumors with *de novo* or acquired resistance. In an effort to investigate the efficacy of lurbinectedin, a new DNA binding drug, Vidal et al. generated serous PDX models by engrafting primary tumor tissue directly onto the mouse ovary surface (36). They included tumors with cisplatin sensitivity, as well as a tumor selected for acquired cisplatin resistance by repeated *in vivo* exposure. They reported a high correlation of histo-pathologic features between the patient and the platinum-sensitive and -resistant PDX tumors. The platinum-sensitive PDX displayed a dose-dependent response to cisplatin treatment, characterized by significant tumor volume reduction. Interestingly, 30–50% of treated mice relapsed at 6 months following treatment, and histo-pathologic features of the relapsed tumors were similar to the un-treated xenografts (36). As expected, cisplatin treatment did not significantly inhibit tumor growth in the cisplatin-resistant PDX model compared to control-treated mice. However, lurbinectedin treatment alone significantly decreased tumor growth in both cisplatin-sensitive and -resistant PDX models, and lurbinectedin in combination with cisplatin was more effective than either drug alone (36). Additional studies demonstrated an increase in apoptosis and mitotic catastrophe in lurbinectedin-treated PDX mice, providing further insight into its mechanism of action. Thus, drug-resistant PDX models can be used to identify therapies that may be effective in patients with tumors resistant to standard agents.

In addition to subcutaneous and intra-peritoneal (IP) engraftments, OC PDX models have been established in mice by sub-renal capsule xenografts, allowing for follicle maturation. Lee et al. have demonstrated a high take-rate (95%) in sub-renal capsule PDX models, including low- or moderate-grade OC tissues that are typically difficult to engraft in subcutaneous or IP models (37). The authors compared histo-pathologic features in the original patient tumor, pre-graft tissue, and post-graft tissue and found no architectural or cytological differences, nor any major differences in immunomarker expression including CK20, CK7, or WT-1 (87–91% overall concordance). This group then investigated five individual sub-renal PDX models for drug response and genetic stability over subsequent passages (38). The authors analyzed the primary tumor and corresponding PDX by array CGH and reported similar gene copy numbers, with the primary tumors consistently clustering with their matching PDX. Furthermore, there was no significant difference in copy number changes between the primary tumor and corresponding PDX (38). These findings further support the accurate reflection of the patient tumor in PDX models. Furthermore, the high engraftment rate of sub-renal capsule PDX models may provide the opportunity to investigate the differences in tumor progression between low- and high-grade ovarian tumors.

As most HG-SOC tumors present at advanced stage, following peritoneal dissemination, IP PDX models are useful for investigation of tumor progression and metastasis. Bankert et al. generated IP PDX models from five different OC patients to examine metastasis and the microenvironment of human ovarian tumors (39). In these mice, tumor growth and spread reflect the patterns that occur clinically whereby tumors grew on surfaces within the peritoneal cavity including the omentum, spleen, ovaries, pancreas, and liver. In addition, these PDX mice formed distended abdomens with ascites fluid containing viable tumor cells, and CA-125 was present in their ascites and blood. Thus PDX OC models not only accurately reflect the histo-pathologic features of the tumor, but also present with clinically relevant disease, making them excellent models to investigate tumor progression.

Patient-derived xenograft models generated from patient ascites may also be useful for investigation of tumor progression and metastasis, and are readily transplanted. Ascites-derived PDX models have been used to characterize genome-wide chromosomal aberrations in *BRCA1*-mutated tumors (40). In addition, Stewart et al. generated ascites-derived PDX models to identify and characterize ovarian TICs (21), and found that these cells are molecularly heterogeneous across different tumors. Direct comparisons of PDX models generated from primary tumors and associated ascites would be helpful to determine whether ascites-derived PDX models accurately reflect the heterogeneity of the solid tumor. Not all patients develop ascites during their clinical course, suggesting that PDX derived only from ascites may not reflect the full disease spectrum.

### PDX models to test targeted therapy

A targeted therapy currently under clinical investigation in OC treatment is inhibition of poly(ADP-ribose) polymerase (PARP), which targets cells with HR defects. PARP inhibition leads to accumulation of single-strand breaks, which generates double-strand breaks in DNA at replication forks. While double-strand breaks are effectively repaired in normal cells by HR repair, cells with deficiencies in *BRCA1/2* use error-prone mechanisms resulting in chromosomal instability and cell death (41). Germline mutations in *BRCA1/2* are present in 17% of HG-SOC cases (42) and in 25% of HG-SOC patients under the age of 50 (43). In addition, loss of BRCA function by genetic or epigenetic processes has been reported in 50% of HG-SOC cases (44). This high frequency of *BRCA* deficiency makes HG-SOC patients ideal candidates for PARP inhibition. PARP inhibitors have been shown *in vitro* and in Phase I/II clinical trials to be an effective treatment in some *BRCA*-deficient tumors, although it is still unclear why all patients with *BRCA1/2* mutations do not respond to PARP inhibitors (45). Furthermore, a proportion of those who do respond eventually progress and thus studies are still necessary to determine the mechanism of resistance to PARP inhibitors. HG-SOC PDX models, generated from tumors before and after treatment, are extremely useful to better understand the mechanisms of therapeutic response and resistance.

The Wang group further analyzed two of their sub-renal PDX models that carried alterations in *BRCA1* and *BRCA2* (38). In one case, DNA sequencing revealed a germline mutation in exon 2 of *BRCA1*, as well as loss of heterozygosity. In the other case, promoter hypermethylation of *BRCA1* was identified in the primary tumor, as well as a sequence variant in intron 2 of *BRCA2*. All of these alterations were maintained in the xenograft tumor. In assessing drug response in these models, tumor volume was decreased in carboplatin/paclitaxel-treated versus control-treated mice; however treatment with the PARP inhibitor PJ34 did not affect tumor growth in the *BRCA* models, despite decreased PAR expression in these tumors (38). It is unclear whether the lack of response was due to poor potency of this PARP inhibitor or due to additional tumor biology causing PARP inhibitor resistance.

In a more recent study to investigate targeted therapy, Kortmann et al. established PDX models from a *BRCA* wild-type and a *BRCA2* germline-mutated HG-SOC tumor to examine response to the PARP inhibitor, olaparib (46). First generation (T1) xenografts were analyzed following daily olaparib treatment of 50 mg/kg for 4 weeks. Immunohistochemical analysis of the *BRCA2* mutated model demonstrated decreased tumor cell proliferation and increased numbers of dead cells following olaparib treatment, while wild-type tumor characteristics were not affected. Moreover, olaparib treatment significantly decreased tumor volume in the *BRCA2* mutant PDX while having no effect on the *BRCA* wild-type PDX (46). These studies demonstrate the value of PDX models for characterizing response to targeted therapy.

In addition to PARP inhibitors, signaling molecules make attractive targets to inhibit tumor growth in HG-SOC and other cancers. The Hedgehog (Hh) pathway promotes proliferation, regeneration, and differentiation in adult somatic tissues, and aberrant activation of the Hh pathway is associated with malignant transformation in several cancers. Combination treatment with standard chemotherapy plus Hh pathway inhibitors has been demonstrated to be effective against proliferation in basal cell cancer, medulloblastoma, and small cell lung cancer, amongst others (47). In OC, it has been reported that 20–50% of cases include Hh pathway activation (48). Furthermore, ectopic expression of Hh factors results in increased proliferation and motility of OC cells, while Hh inhibition impairs the growth of OC cell lines *in vivo*. Thus the Hh pathway may be a potential therapeutic target in OC treatment.

McCann et al. further examined the potential of Hh inhibitors as OC treatment using subcutaneous PDX models of serous OC (49). In this study, they treated a serous PDX model reported to have an activated Hh signaling pathway with the Hh pathway inhibitor cyclopamine. The cyclopamine-treated PDX mice had significantly decreased tumor volume compared to control-treated mice. In addition, the authors tested the efficacy of IPI-926, a derivative of cyclopamine that has increased oral bioavailability and potency and is currently in Phase I/II clinical trials, alone and in combination with standard first-line chemotherapy. In three different serous PDX models with activated Hh signaling pathways, Treatment with IPI-926 alone, or in combination, resulted in decreased tumor growth similar to results from chemotherapy alone, compared to controls (49). Interestingly, when the PDX mice were maintained on IPI-926 alone following combination treatment, tumor regression was retained for up to 50 days following initial therapy. These studies indicate the utility of PDX models in examining combination therapy as well as maintenance therapy, in clinically relevant models.

### PDX cohorts

One of the most useful features of PDX models is their renewability, providing a repository of xenografts, tissues, and cell lines for researchers to access, along with relevant clinical and molecular data (Table 1). These cohorts provide fully annotated, genome-specific PDX models as training and test sets, providing the opportunity to efficiently bring molecular targeted drugs into clinical trials for the treatment of OC. In order for these cohorts to be fully beneficial, comprehensive annotation is essential, particularly two major characterizations: (a) the methods utilized to generate and maintain the PDX models (e.g., source tissue to confirm HG-SOC origin, fragmentation or mincing versus digestion to limit selection and possibly allow retention of infiltrating stroma, no prior *in vitro* culture to ensure reflection of primary tumor, method of implantation, etc.); (b) histotype, molecular, and genomic characterization (mutation, gene expression, CGH, CNV analysis), as well as response to standard therapy, of the PDX models (Table 1). Upon complete characterization and annotation, this resource will greatly accelerate the development of newly targeted therapies and identification of predictive biomarkers in OC, further bridging the gap between laboratory-based discoveries of novel therapeutic targets and clinical care.

**Table 1:** **Summary of ovarian cancer PDX models**.

Reference	Histotype (*n*)	Culture	Method	Treatment	Molecular annotation
Repasky group (31–33)	Serous (14), endometrioid (1), mucinous (2), clear cell (1), unspecified (2)	No prior *in vitro* culture	Minced, implanted on GFP or SC, SCID mice	IL-12, Flt-3 ligand (in serous models)	Not reported
Ghamande et al. (34)	Serous (6)	No prior *in vitro* culture	Minced, implanted SC, SCID mice	CD40 ligand, cisplatin, paclitaxel	Not reported
Kolfschoten et al. (35)	Serous (5), mucinous (4), clear cell (2), undifferentiated (3), carcinosarcoma (1)	No prior *in vitro* culture	Fragmented, implanted SC, athymic nude mice	Cisplatin, cyclophosphamide, doxorubicin, hexamethylmelamine, methotrexate, 5-fluorouracil	Glutathione content and glutathione-dependent enzyme activity
Vidal et al. (36)	Serous (1)	No prior *in vitro* culture	Implanted on ovary surface, athymic nude mice	Lurbinectedin (PM01183), cisplatin	Not reported
Wang group (37, 38, 44)	Serous (6), mucinous (2), granulosa cell tumor (2), leiomyosarcoma (1), clear cell (1), unspecified (1)	No prior *in vitro* culture	Fragments implanted sub-renal, NOD/SCID mice	Carboplatin, paclitaxel, PARP-1 inhibitor (PJ34) (in *BRCA1* null model)	CGH, *BRCA1/2* mutations (three serous, one clear cell, one leiomyosarcoma)
Bankert et al. (39)	Serous (4), undifferentiated (1)	No prior *in vitro* culture	Aggregates injected IP, NSG mice	IL-12	Not reported
Kortmann et al. (46)	Serous (2)	No prior *in vitro* culture	Fragments implanted sub-renal, NOD/SCID mice	Olaparib, carboplatin	*BRCA1/2* mutations, copy number
McCann et al. (49)	Serous (4)	No prior *in vitro* culture	Single cell suspensions injected SC, NOD/SCID mice	Cyclopamine (1), Hedgehog inhibitor (IPI-926), paclitaxel, carboplatin (3)	Not reported
Hylander et al. (50)	Serous (2)	No prior *in vitro* culture	Fragments implanted SC, SCID mice	Not reported	Stromal annotation by IHC
Stewart et al. (21)	Serous (31 primary tumor or ascites)	No prior *in vitro* culture	Digested, single cell suspensions injected as 1:1 HBSS:Matrigel in mammary fat pad, NOD/SCID mice	Not reported	Tumor-initiating cell markers CD133, CD44, CD117, EpCAM, ALDH1
Indraccolo et al. (40)	Serous (2 ascites)	No prior *in vitro* culture (T1), 2–3 passages (T2)	Ascitic fluid collected at recurrence, IP injection in SCID mice	Not reported	*BRCA1* mutations and expression, LOH, chromosomal aberrations by MLPA

*GFP, gonadal fat pad; SC, subcutaneous; IP, intra-peritoneal; NSG, NOD-SCID IL2Ry^−/−^; MLPA, multiplex ligation-dependent probe amplification*.

## Limitations

Probably the most noted limitation of PDX models involves the use of immunocompromised mice, which may attenuate the impact of the tumor microenvironment on tumor growth and drug response. In addition, stromal components such as vasculature or secreted stromal factors are increasingly being targeted by novel therapies. Thus, it is imperative that PDX models recapitulate the heterogeneity of the patient tumor in order to accurately test these novel therapies. In a recent study, Hylander et al. investigated vascularization and stromal formation in 37 subcutaneous PDX models in SCID mice created from a range of tumor types, including ovary, pancreas, kidney, and colon cancers (50). Successfully engrafted tumors were histologically examined for stromal factors and blood vessels. Their findings demonstrated that at the first passage (15–25 weeks), tumors no longer contained human stromal factors or vasculature; indeed, the stromal fibroblasts and vessel markers within the tumor were of murine origin (50). Kinetic studies suggested that loss of human vascularization markers occurred within 3–9 weeks, depending on tumor type. The authors conclude that in PDX models in which tumors are engrafted directly into immunocompromised mice, tumor growth is supported by host stroma and vasculature, suggesting that studies of therapies targeting human stromal components may not be adequate in these models.

To overcome these challenges, various approaches have been applied and additional models have been generated with the aim of recapitulating the tumor microenvironment. Engraftment of whole, non-disrupted chunks of human tumor helps to preserve tumor microenvironment components including leukocytes, fibroblasts, extracellular matrix, and vasculature (51). In addition, the use of NOD-SCID IL2Rγ^−/−^ mice provides improved PDX models for tumor–stromal interactions as they maintain tumor-associated leukocytes and stromal fibroblasts for up to 9 weeks after implantation (52). In the previously described IP OC PDX study by Bankert et al. performed in NOD-SCID IL2Rγ^−/−^ mice, the authors identified functional human lymphocytes and fibroblasts in tumors from multiple organs within the peritoneal cavity up to 177 days following engraftment (39). These findings suggest that future PDX models would provide greater value if generated in NOD-SCID IL2Rγ^−/−^ mice, particularly for studies involving drug response or microenvironment-targeting treatments.

## Conclusion

The establishment of PDX models that recapitulate the complexity and genetic heterogeneity of HG-SOC will guide personalized cancer therapy and be invaluable toward establishing research priorities and strategies for developing new and more effective approaches to treatment in patients with recurrent OC. A repository of extensively characterized HG-SOC PDX models can be used for drug screening and discovery as well as biomarker development and testing. Furthermore, PDX models generated at initial diagnosis as well as at the time of recurrence will not only permit personalized treatment options, but in the long-term serve to enrich the recruitment and accrual of patients into early phase clinical trials.

## Authors Contribution

Clare L. Scott, Marc A. Becker, Paul Haluska, and Goli Samimi all contributed to the writing and editing of the manuscript.

## Conflict of Interest Statement

The authors declare that the research was conducted in the absence of any commercial or financial relationships that could be construed as a potential conflict of interest.
